# Stability indicating potentiometric method for the determination of palonosetron HCl using two different sensors

**DOI:** 10.1038/s41598-022-17349-y

**Published:** 2022-07-28

**Authors:** Mahmoud A. Tantawy, Dalia A. Elshabasy, Nadia F. Youssef, Sawsan M. Amer

**Affiliations:** 1 Analytical Chemistry Department, Faculty of Pharmacy, Cairo University, Cairo, Egypt; 2 Chemistry Department, Faculty of Pharmacy, October 6 University, 6 October City, Giza Egypt; 3 Egyptian Drug Authority, Giza, Egypt

**Keywords:** Analytical chemistry, Electrochemistry

## Abstract

This paper presents a novel potentiometric approach for the determination of palonosetron HCl using two sensors; ionophore-free and ionophore-doped ones. The two sensors successfully determined the cited drug in the range of 1 × 10^–5^–1 × 10^–2^ M with respective Nernstian slopes of 54.9 ± 0.25 and 59.3 ± 0.16 mV/decade. Incorporating calix[8]arene as an ionophore resulted in a lower detection limit (LOD = 3.1 × 10^–6^ M) and enhanced selectivity when compared to the ionophore-free sensor (LOD = 7.9 × 10^–6^ M). This modification was also associated with faster response for the ionophore-doped sensor (response time = 20 s) compared to the ionophore-free one (response time = 30 s). The two sensors showed a stable response over a pH range of 3.0–8.0. They successfully determined palonosetron HCl in presence of its oxidative degradation products. They were also used for direct determination of the drug in commercially available parenteral solution without any interference from other dosage forms’ additives.

## Introduction

Palonosetron HCl (PALO), chemically designed as (S)-2-[(3S)-Quinuclidin-3-yl]-2,3,3a,4,5,6-hexahydro-1H-benzo[de]isoquinolin-1-onehydrochloride^1^, is a highly selective and potent second generation serotonin (5-HT_3_) receptor antagonist. It is characterized by strong binding affinity and long plasma elimination half-life. PALO shows efficacy in preventing nausea and vomiting resulting from highly emetogenic chemotherapy^2^. The drug is official only in United States Pharmacopeia (USP)^3^ where it is determined by a reversed-phase high performance liquid chromatographic (HPLC) method using acetonitrile: water: trifluoroacetic acid (280: 720: 0.67, v/v/v) as a mobile phase.

Literature survey revealed various analytical methods for PALO determination, including spectrophotometric^4^, high performance thin layer chromatographic (HPTLC)^5–7^ and HPLC ones^7–19^. According to previously conducted stability study by our research group, the drug was found to be susceptible to oxidation where two stability-indicating chromatographic methods were developed^7^. Although all these reported methods are suitable for PALO determination, they have two main disadvantages; (i) expensive equipment needed, and (ii) tedious sample preparation steps.

The simplicity, sensitivity and selectivity associated with potentiometric field have promoted its application in different types of samples; biomarkers, inorganic ions and pharmaceuticals analysis^20–26^. This potentiometric technique depends on presence of an ionizable function group in the analyte of interest leading to its selective partitioning to a plasticized lipophilic membrane in which suitable ion-exchanging salts as well as complexation agents (ionophores) are incorporated for such purpose^27^. These lipophilic membranes are widely utilized with different substrates to construct disposable ion-selective electrodes (ISEs)^21,22,28^.

Among the mostly widely used ionophores are calixarenes which are chemically consisting of phenol units linked via alkylidene groups. This provides them their unique cavity-shaped configuration that leads to formation of typical host–guest complexes with different compounds depending on some factors, such as; cavity-size, conformation and substituents. In nutshell, calixarenes are suitable for a variety of applications in ISEs fabrication^22,29,30^.

Here, we develop the first ISE for the potentiometric determination of PALO. We also exploit the advantages of selectivity enhancement, related to calix[8]arene incorporation in potentiometric ISE, in order to demonstrate its use as a stability-indicating one. Two ISEs are fabricated and their performance characteristics are compared; one without incorporation of ionophore (ionophore-free senor), and another one utilizing calix[8]arene as an ionophore (ionophore-doped senor).

## Results and discussion

Ion-selective membranes are usually prepared from PVC, electroactive substance (ion-association complex) and a plasticizer. The role of PVC is to provide an inert solid support structure in which the rest of components are embedded. The plasticizer dissolves the ion association complex, plasticizes the membrane and affects the lipophilicity of PVC membrane. It also alters the distribution coefficient (K) of different species thus affecting the performance characteristics of electrode^31^*.* In potentiometric applications, selectivity and sensitivity are the main aim that guide optimization plan during method development. A key component in ISE fabrication that can significantly improve selectivity and sensitivity is doping the PVC polymeric membrane with an ionophore^32^*.* The response of membranes containing ionophores is largely governed by molecular recognition where the analyte functions as the guest and the ionophore plays the role of host^33^. ISEs containing ionophores have been shown to increase the selectivity of the sensor toward the detection of specific analytes^27^. Calixarenes are widely used as ionophores for various ions via dipole–dipole interactions where they can make stable host–guest inclusion complexes with different types of cation substrates. As a result, they have been largely exploited for the development of a number of ISEs^22,29,34^.

### Stability study

As per our previously reported work, PALO was subjected to forced acidic, alkaline, oxidative, photolytic and thermal degradation conditions^7^. Observed degradation was noticed only under oxidative condition upon refluxing with 6% H_2_O_2_ for 6 h. Three degradation products were separated on HPTLC plate and mass analysis was then conducted by means of Advion compact mass spectrometer^7^. The obtained mass spectra of PALO along with its three oxidative degradation products are shown in Figure S1, supplemental information. As a result and in this work, calix[8]arene was incorporated as an ionophore and the fabricated ISE was compared to the ionophore-free one in terms of sensitivity and selectivity for PALO determination in presence of its oxidative degradation products. The pKa value of PALO is ≈ 8.81, so at pH 5.0, the studied drug has a positive charge. The use of TPB as counter ion for the cationic PALO in the two proposed ion-sensitive membrane sensors was suggested.

### Response characteristics of the proposed sensors

**Figure 1:**
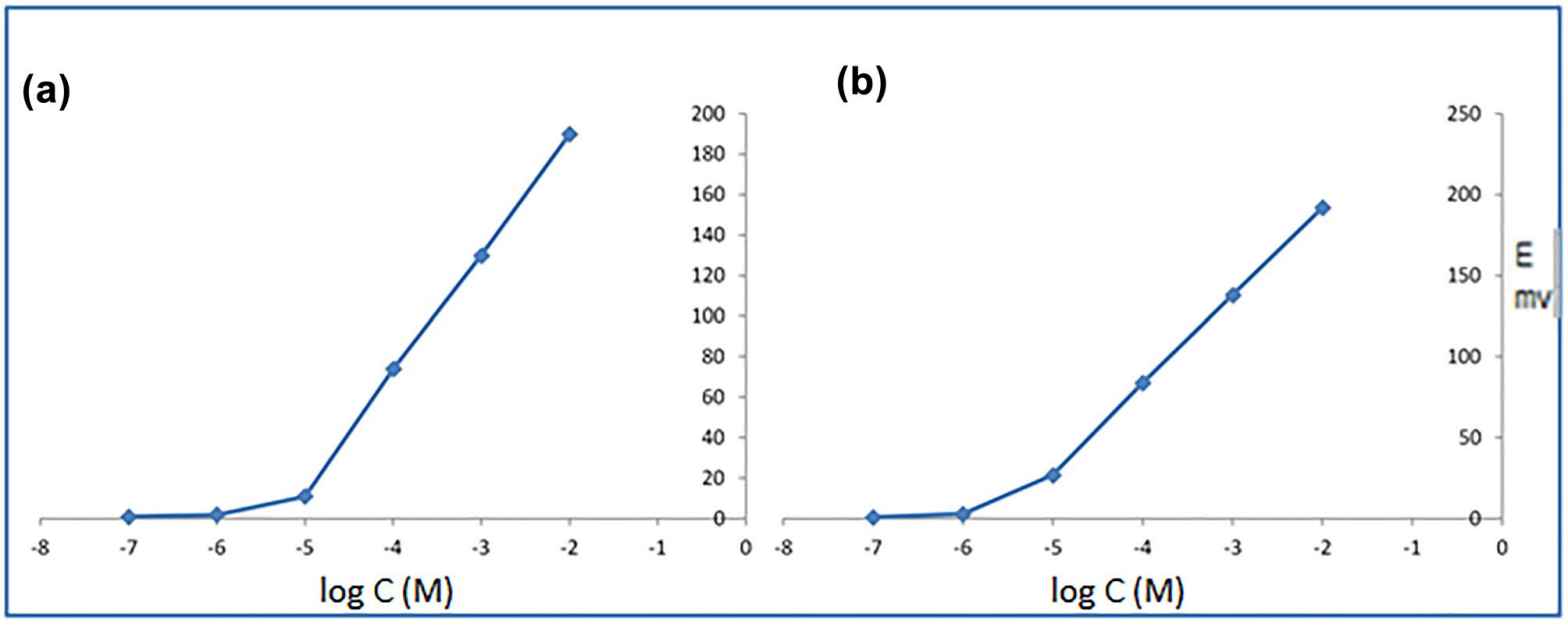
Profile of the potential in mV against log concentration of PALO at pH 5.0 for (**a**) ionophore-free sensor and (**b**) ionophore-doped sensor.

The performance characteristics of the two proposed ISEs were assessed according to the IUPAC standards^27^, Table 1. Two calibration curves were constructed, showing the same linearity range; 1 × 10^–5^ to 1 × 10^–2^ M as displayed in Fig. 1. The obtained slopes were 54.9 ± 0.25 and 59.3 ± 0.16 mV/decade for ionophore-free and ionophore-doped sensors, respectively, with respective detection limits of 7.9 × 10^–6^ and 3.1 × 10^–6^ M. The enhanced responses of ionophore-doped sensor are attributed to; (1) presence of rigid calix[8]arene cavity which selectively complex with PALO, (2) fast complexation-decomplexation kinetics for reversible transduction processes, and (3) high lipophilicity (especially with 8 phenolic units of calix[8]arene) preventing complexed PALO from leaching from nonpolar membrane into aqueous phase^35^.

**Table 1:** Electrochemical response characteristics of the investigated PALO sensors.

Parameter	Ionophore-free sensor	Ionophore-doped sensor
Slope (mV/decade)^a^	54.9	59.3
Intercept (mV)	302.4	308.8
LOD (mol/L)^b^	7.9 × 10^–6^	3.1 × 10^–6^
Regression coefficient	0.9995	0.9998
Response time (s)	30	20
Temperature	25 °C	
Working pH range	3.0–8.0	
Concentration range (M)	1.0 × 10^–5^–1.0 × 10^–2^	
Stability (days)	20	30
Repeatability^c^	0.68–0.82	0.52–0.79
Intermediate precision^d^	1.06–1.85	0.87–1.02
Accuracy (R %)	99.69	100.04
Emegrand vial 0.25 mg/5 mL (Mean ± RSD%)^e^	101.50 ± 1.7	102.14 ± 0.5

^a^Average of four determinations.

^b^Limit of detection calculated at the interception of extrapolated arms in potential profile.

^c^The intraday (n = 3) RSD% of concentrations 10^–2^, 10^–3^ and 10^–4^ M PALO.

^d^The interday (n = 3) RSD% of concentrations 10^–2^, 10^–3^ and 10^–4^ M PALO.

^e^The average of five determinations.

### Effect of pH

**Figure 2:**
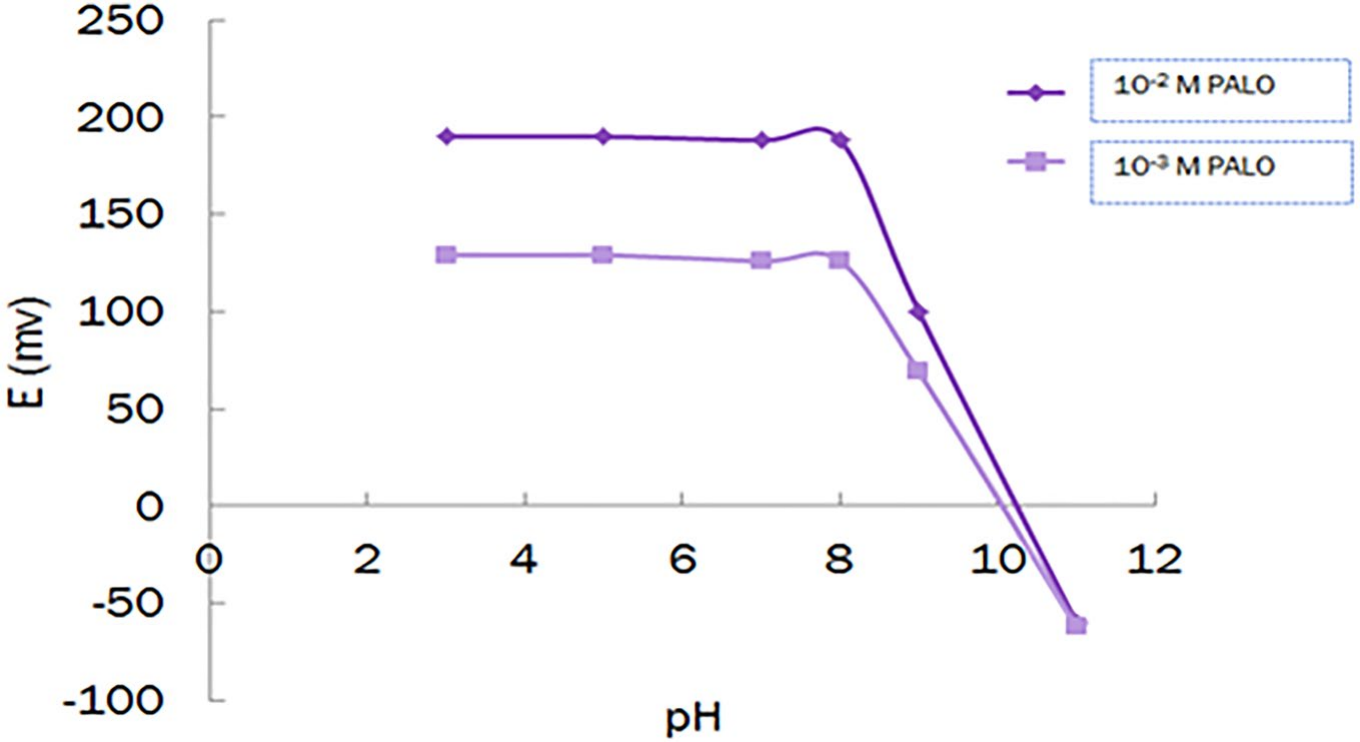
Effect of pH on the performance of ionophore-free sensor.

The effect of pH on the response of the proposed sensor is investigated by using 1 × 10^–2^ M and 1 × 10^–3^ M PALO standard solutions at different pH values, ranging from 3.0–11.0. The obtained potentials were recorded at each pH value. It was found that the investigated electrode showed a stable response over a pH range of 3.0–8.0, Fig. 2.

### Effect of temperature

**Figure 3:**
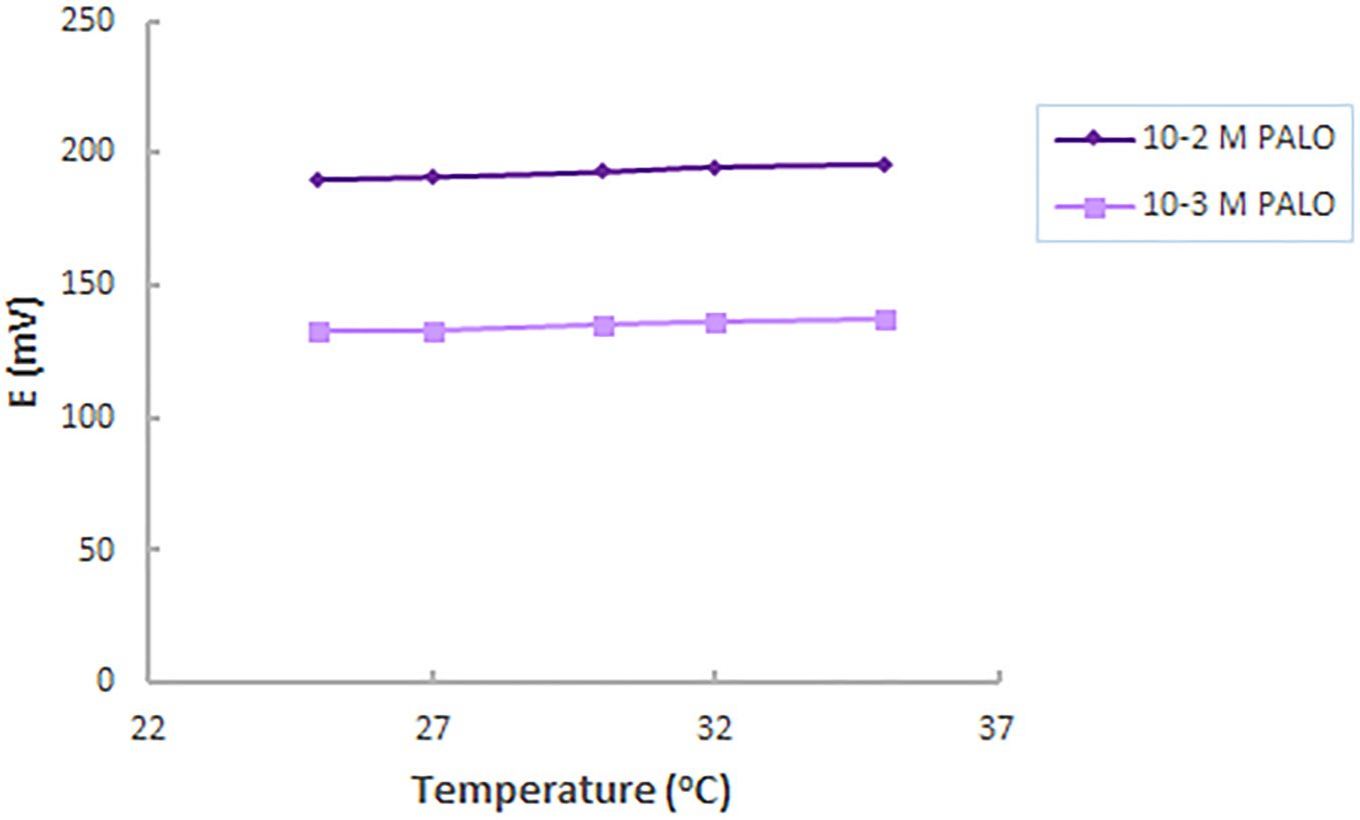
Effect of temperature on the performance of ionophore-free sensor.

The effect of temperature on the response of the proposed ISE was studied. 1 × 10^–2^ M and 1 × 10^–3^ M PALO standard solutions were used whereas a fairly stable response is observed upon increasing temperature in the range of 25–35 °C. These results indicate reasonable thermal stability of the proposed electrode up to 35 °C, Fig. 3.

### Selectivity of the proposed sensors

**Figure 4:**
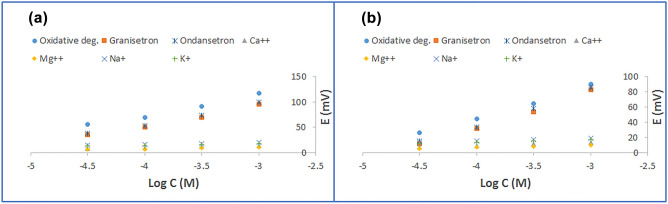
The responses of (**a**) ionophore-free sensor and (**b**) ionophore-doped sensor as function of log concentration for some inorganic ions, PALO oxidative degradation products and PALO structurally related drugs (granisetron & ondansetron) in selectivity measurements.

Selectivity of proposed PALO sensors in presence of interfering ions was evaluated by separate solutions method. Four inorganic ions; Na^+^ (as citrate), K^+^, Ca^2+^ and Mg^2+^ (as chlorides), oxidative degradation products as well as structurally related granisetron were selected for this study. The two proposed sensors showed non-Nernstian responses to the inorganic ions, Fig. 4. This is attributed to the hydrophobic nature of ion selective membrane which hinders the hydrophilic inorganic ions exchange. As a result, no need to determine selectivity coefficients for those ions^36^. On the other hand, oxidative degradation products and structurally related drugs (granisetron & ondansetron) showed near-Nernstian responses to monovalent cations (≈ 45 mV/decade) over the considered range, Fig. 4. Selectivity coefficients were consequently calculated, Table 2. As shown, about one order of magnitude enhancement was noticed for ionophore-doped sensor. This supports its exceedances over ionophore-free one as stability-indicating sensor. In nutshell, incorporation of calix[8]arene, during fabrication of ISE, improved the selectivity of the proposed ionophore-doped sensor as compared to the ionophore-free one.

**Table 2:** Potentiometric selectivity coefficient (${\mathrm{K}}_{\mathrm{PALO},\mathrm{Int}}^{\mathrm{pot}}$) for the investigated PALO sensors using the separate solutions method.

Interferent	Selectivity coefficient for ionophore-free sensor^a^	Selectivity coefficient for ionophore-doped sensor^a^
Oxidative degradation products	6.0 × 10^–1^	7.6 × 10^–2^
Granisetron	2.2 × 10^–1^	4.1 × 10^–2^
Ondansetron	2.6 × 10^–1^	4.5 × 10^–2^

^a^Average of three separate determinations.

### Application to dosage form

The two proposed sensors were successfully applied for the determination of PALO in Emegrand vial, Table 1. It is worth noting that this potentiometric method offers the advantage of direct PALO determination without any pretreatment steps.

### Statistical analysis and sensors evaluation

The results obtained for the potentiometric analysis of PALO were statistically^37^ compared with those obtained by applying an official HPLC method^3^. The calculated values of t and F are less than their corresponding tabulated ones, which reveals that there is no significant difference between the suggested and official method with respect to accuracy and precision, Table 3. Moreover, a point-by-point comparison with the two reported stability-indicating chromatographic methods was conducted, Table 4. Besides the no need for preliminary drug preparation, our proposed sensors achieved a comparable quantification limit (LOQ) as well as ability of PALO determination in presence of other structurally related drugs.

**Table 3:** Statistical comparison between the results obtained by the proposed ISE potentiometric method and the official method for determination of PALO in its pure form.

Parameter	ISE potentiometric method^a^		Official method^b^
	Ionophore-free sensor	Ionophore-doped sensor	
Mean	99.69	100.04	100.33
SD	0.65	0.35	0.38
SE	0.21	0.12	0.22
n	9	9	3
Variance	0.42	0.12	0.14
Student’s t-test	2.10 (2.23)^c^	1.16 (2.23)^c^	NA
F-test	3 (19.37)^c^	1.17(4.46)^c^	NA

^a^9 determinations of 3 concentration levels by the proposed ISE method.

^b^3 determinations of 3 concentrations by USP 41, HPLC–UV method for PALO.

^c^The values in parentheses are the corresponding tabulated values of t and F at p = 0.05.

**Table 4:** An overview on the reported stability-indicating chromatographic methods compared to the proposed potentiometric method for the determination of PALO.

Reference No		LOQ	Time^a^	Application
^7^	HPLC	0.1 µg mL^−1^	10 min	Dosage form In presence of degradation products
	HPTLC	0.1 µg band^−1^	20 min	
This work		1.0 × 10^–5^ M	20 s	Dosage form In presence of degradation products In presence of structurally related drugs; granisetron & ondansetron

^a^Time required for acquiring data from one sample measurement.

## Conclusions

This work demonstrates the first potentiometric method for palonosetron HCl determination. Two ion-selective electrodes were fabricated; one without incorporation of ionophore (ionophore-free senor) and the second one utilizing calix[8]arene as an ionophore (ionophore-doped senor). The incorporation of calix[8]arene, as an ionophore, improved the limit of detection as well as the selectivity of the proposed sensor towards the most likely formed degradation products and the structurally related drugs. Selectivity assessment revealed that the fabricated sensors could be applied as stability-indicating ones and in direct determination of the cited drug in presence of its dosage form additives. The proposed method was found to be sensitive, rapid, easy to use, selective, simple and more economic for palonosetron HCl determination as compared to other reported ones. The method showed also good applicability for determination of the cited drug in its marketed dosage form (Emegrand vials) promoting its use in different quality control laboratories.

## Methods

### Materials and reagents

Palonosetron hydrochloride working standard (99.29%) was supplied from National Organization for Drug Control and Research (NODCAR, Giza, Egypt).

Commercially available Emegrand (0.25 mg/5 mL) vial for I.V., batch no.:171260, (Delta Grand Pharma, Egypt) was purchased from the Egyptian market.

Oxidative degradation products were prepared following our reported protocol^7^. Briefly, the intact drug was refluxed with 6% H_2_O_2_ for 6 h. The solution was then evaporated and the resulted residue was dissolved in methanol.

Sodium tetraphenyl borate (TPB) [Sigma-Aldrich, Steinheim, Germany], polyvinylchloride (PVC) [Fluka Chemie GmbH, St. Louis, USA], nitrophenyl octyl ether (NPOE) [Fluka Chemie GmbH, St.Louis, USA], tetrahydrofuran (THF) [BDH, Poole, England], calix[8]arene [Sigma-Aldrich, Steinheim, Germany] and double distilled deionized water [Otsuka, Cairo, Egypt].

The Britton-Robinson buffers^38^ in the pH range of 2.0–12.0 were prepared by mixing equal volumes of 0.04 M acetic acid, 0.04 M boric acid and 0.04 M phosphoric acid. The required pH values were adjusted using 0.2 M NaOH standard solution.

### Instrumentation

Ag/AgCl reference electrode (Thermo Scientific Orion 90–02, MA, USA). pH meter for pH adjustments and potential measurements (Hanna 211). Magnetic stirrer (Bandelin Sonorex, Rx 5105). HPTLC aluminum plates 10 × 10 cm precoated with 0.25 mm silica gel 60 F_254_ (Merck, Germany) were used for stability study. The plates were developed at ambient temperature using a mixture of methanol: ammonia (10: 0.5, v/v) as the developing system in a CAMAG twin-trough chamber previously saturated with the developing system for 30 min. Advion compact mass spectrometer (CMS, USA) provided with ESI ion source was utilized for mass analysis.

### Fabrication of ion-selective electrodes

The ion-selective sensors were prepared by mixing PVC (190 mg), NPOE (0.38 mL) and TPB (5 mg) for preparation of the ionophore-free sensor. PVC (190 mg), NPOE (0.38 mL), TPB (5 mg) and Calix[8]arene (10 mg) for preparation of ionophore-doped one. The membrane components were dissolved in THF (6.0 mL), and poured into Petri dishes which were then covered with filter papers and left to stand overnight at room temperature allowing the THF to evaporate. Membranes with a thickness of 0.1 mm were obtained where 8-mm diameter disks were cut using a cork borer. Disks were then affixed to a PVC tips using THF and affixed onto the end of a glass body electrode. The electrodes were then filled with an inner-filling solution (IFS) with equal volumes of 10^–4^ M PALO and 10^–4^ M KCl. A Ag/AgCl wire was placed in the IFS and served as an internal reference electrode. Each sensor was conditioned by placing them in a solution containing 10^–4^ M PALO for 24 h prior to use and was stored in the same solution when not in use.

### Sensors calibration

**Figure 5:**
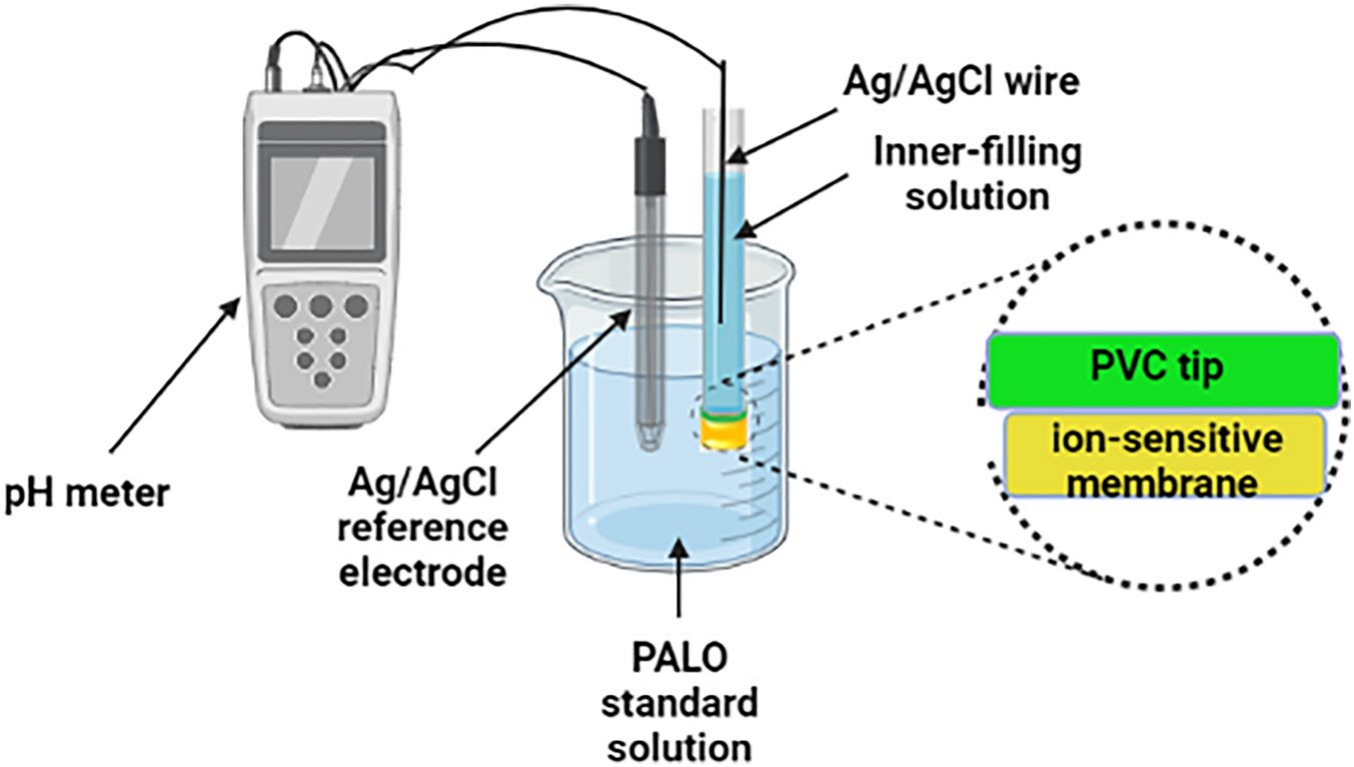
A diagram for measurement process using the proposed method. Figure 5 was created using BioRender (https://biorender.com).

Calibration of the conditioned PALO sensors is performed by immersing them separately in conjunction with double junction Ag/AgCl reference electrode in different PALO standard solutions (1 × 10^–5^ to 1 × 10^-2^ M) prepared in buffer pH 5.0. The ISEs were allowed to equilibrate while stirring and potential difference (emf) readings were the recorded (within ± 1 mV). The membrane sensors were washed between measurements with the buffer. The recorded potentials were finally plotted as a function of logarithm PALO concentrations in buffer pH 5.0 at 25 °C. A diagram for measurement process is shown in Fig. 5.

### Selectivity studies

The potentiometric selectivity coefficients (${K}_{PALO,Int}^{pot}$) of the proposed ISEs were evaluated according to IUPAC^27^ guidelines using the separate solutions method^39,40^. Calibration curves for some inorganic cations, oxidative degradation products as well as a structurally related organic ions, granisetron and ondansetron, were constructed using the two proposed sensors. Potentials for the same concentration (1 × 10^–4^ M) of PALO cation and interfering ions were measured separately, and the rearranged Nicolsky–Eisenman equation was applied^36,39^.

$$Log{K}_{A,B}^{pot}=\left[\frac{E_{B}-E_{A}}{\left(\frac{2.303RT}{Z_{A}F}\right)}\right]+\left(1-\frac{Z_{A}}{Z_{B}}\right)\mathrm{log}\,[A]$$

where E_A_ and E_B_ are potentials measured for ion of interest (with Z_A_ charge) and interfering ion (with Z_B_ charge), respectively, and (2.303RT/Z_A_F) is the slope of the calibration curve in mV/decade.

### Application to pharmaceutical dosage form

Five vials of Emegrand were emptied and transferred into a 50-ml volumetric flask. The volume was completed to the mark with Robinson buffer pH 5.0 to prepare a solution of 8.4 × 10^–4^ M PALO. The emfs produced by immersing the prepared electrode in conjunction with the double junction Ag/AgCl reference electrode in the prepared solution at 25 °C were recorded. The concentration of PALO was calculated from the following regression equations:

$${\text{Y}} = {54}.{\text{9X}} + {3}0{2}.{\text{4 For ionophore - free sensor}}$$

$${\text{Y}} = {59}.{\text{3X}} + {3}0{8}.{\text{8 For ionophore - doped sensor}}$$

where Y is the potential in mV and X is the logarithm of the concentration in M.

## Supplementary Information

Supplementary Information.

## Data Availability

The data that support the findings of this study are available from the corresponding author upon reasonable request.
