# Supplementary material for: Stability indicating potentiometric method for the determination of palonosetron HCl using two different sensors
Source: Sci Rep. 2022 Jul 28;12:12966. doi: 10.1038/s41598-022-17349-y (PMC9334296; doi:10.1038/s41598-022-17349-y)
Supplement: Supplementary file 1 — Supplementary Information. [file 41598_2022_17349_MOESM1_ESM.docx]

Supplemental Information

For

**Exploiting the advantages of ionophore incorporation in stability-indicating potentiometric determination of palonosetron HCl**


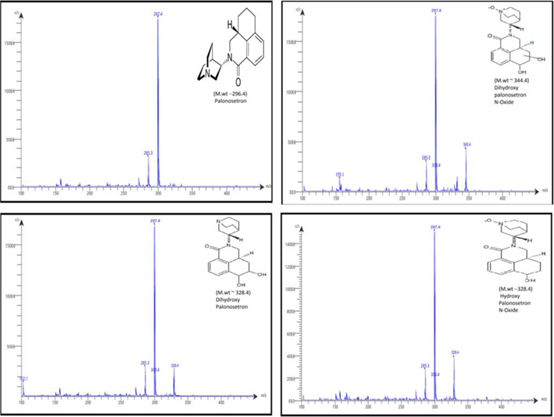


A

B

D

C

**Figure S1**. TLC–MS spectra of; palonosetron (A) along with its three oxidative degradation products (B, C & D). Inset; their chemical structures.
